# Effects of Berberine on Growth Performance, Serum Biochemical Parameters, Hepatic Antioxidant Capacity and Metabolism in *Monopterus albus*

**DOI:** 10.3390/life16050829

**Published:** 2026-05-17

**Authors:** Xinran Tao, Weiwei Huang, Yifan Zhao, Muyan Li, Yuning Zhang, Hang Yang, Wenzong Zhou, Mingyou Li

**Affiliations:** 1 Key Laboratory of Exploration and Utilization of Aquatic Genetic Resources, Ministry of Education, Shanghai Ocean University, Shanghai 201306, China; xxxholic98@163.com (X.T.); 17854232836@163.com (Y.Z.); limuyan0720@163.com (M.L.); 2 Eco-Environmental Protection Research Institute, Ministry of Agriculture and Rural Affairs, Shanghai Academy of Agricultural Sciences, Shanghai 201403, China; hwwswx@163.com (W.H.); ynzhang@saas.sh.cn (Y.Z.); yhangqu2024@163.com (H.Y.)

**Keywords:** berberine, *Monopterus albus*, growth performance, oxidative stress, hepatoprotective, metabolomics

## Abstract

Intensive aquaculture of rice field eel (*Monopterus albus*) is constrained by oxidative stress induced by high-density culture resulting in growth inhibition, while prophylactic antibiotics pose escalating risks of drug resistance and food safety hazards. This study addresses the critical need for developing efficient, environmentally friendly functional feed additives as sustainable growth promoters in intensive aquaculture. To investigate the dietary berberine (BBR) effect on promoting growth performance, hepatic antioxidant capacity and metabolism in *M. albus*, four experimental groups were established: control (CON, 0 mg/kg) and berberine-supplemented groups (BBR25, 25 mg/kg; BBR50, 50 mg/kg; BBR100, 100 mg/kg). Growth performance, serum biochemical parameters, hepatic antioxidant capacity, and liver metabolomics (LC-MS) were evaluated after the 8-week feeding trial. BBR50 and BBR100 had significantly increased final weight, weight gain rate (WG), and survival rate (SR), while reducing feed conversion ratio (FCR) (*p* < 0.05). Serum glucose (Glc), total cholesterol (TC), triglycerides (TG), and low-density lipoprotein cholesterol (LDL-C) were decreased (*p* < 0.05), while high-density lipoprotein cholesterol (HDL-C) and phosphofructokinase (PFK) activity were increased (*p* < 0.05). Alanine aminotransferase (ALT), aspartate aminotransferase (AST) and alkaline phosphatase (ALP) were significantly reduced (*p* < 0.05). Superoxide dismutase (SOD), catalase (CAT) and glutathione peroxidase (GPx) were upregulated (*p* < 0.05), whereas malondialdehyde (MDA) was downregulated (*p* < 0.05). Metabolomics identified 98 differential metabolites, with significant enrichment of metabolites associated with arachidonic acid metabolism, histidine metabolism, arginine/proline metabolism, tryptophan metabolism, and pathways related to mTOR signaling. Overall, dietary supplementation with 50 mg/kg BBR emerged as a practically favorable dose among the tested concentrations for promoting growth performance and feed utilization efficiency, whereas 100 mg/kg BBR was associated with lipid and amino acid metabolic alterations suggestive of metabolic reprogramming and antioxidant-related shifts, without conferring additional growth benefits.

## 1. Introduction

Since 1961, global per capita consumption of fish and aquatic products has risen continuously, doubling from approximately 9 kg to over 20 kg, with a growth rate exceeding that of most other animal-derived foods [1]. The increasing demand for fish consumption is driven by multiple factors, such as enhanced health consciousness [2]. In response to the continuously increasing demand, fish production has also risen year after year. Since the early 21st century, aquaculture has contributed nearly all the growth in per capita fish supply [3]. To increase production and reduce costs, the aquaculture industry has increasingly adopted intensive aquaculture models in recent years [4]. However, stressors in intensive aquaculture, including overcrowded living conditions, improper management practices, and poor water quality [5], often suppress fish immune function [6], impair metabolic capacity [7], and redirect energy resources from growth to stress responses [8], thereby leading to disease outbreaks, compromising normal development and survival [9], and ultimately exerting negative effects on fish yield and economic performance. In practical production, antibiotics are commonly employed to prevent and treat various aquatic diseases. However, frequent antibiotic use leads to increased drug resistance in cultured species and causes drug residues in aquatic products, undoubtedly elevating food safety risks and potentially disrupting ecological balance through food chain contamination [10,11,12,13]. The diminishing therapeutic efficacy of antibiotics compels farmers to increase dosages and administration frequency, thereby creating a vicious cycle [14,15]. To address this situation, the exploration and development of efficient, environmentally friendly, and safe functional feeds represent a critical priority for healthy fish farming under current industrialized high-density aquaculture conditions. Numerous natural herbs have been utilized to promote the growth of cultured species, including milkvetch root (*Astragalus membranaceus*) [16], licorice (*Glycyrrhiza uralensis*) [17], honeysuckle (*Lonicera japonica*) [18], and garlic (*Allium sativum* L.) [19]. These botanicals are often incorporated directly as crude powdered extracts, a practice that frequently imposes substantial challenges on quality control and regulatory approval [20,21]. Berberine, isolated from the rhizomes of Chinese goldthread (*Coptis chinensis*) [22], can circumvent these limitations.

Berberine, an isoquinoline alkaloid with a precisely characterized chemical structure (C_20_H_18_NO_4_^+^) [23], exhibits broad-spectrum biological activities, including antimicrobial [24], immunomodulatory [25] and metabolic regulatory functions [26]; it can alleviate oxidative stress in organs [27,28]. It also plays a significant protective role in metabolic and inflammatory diseases [26,29]. In aquaculture, berberine has been applied as a functional feed additive with antioxidant, metabolic-enhancing, and growth-promoting properties in grass carp (*Ctenopharyngodon idellus*) [30], yellow catfish (*Pelteobagrus fulvidraco*) [31], and tilapia (*Oreochromis niloticus*) [32].

Rice field eel (*Monopterus albus*) is a famous freshwater aquaculture species in China, with national annual production exceeding 350,000 metric tons [33]. With the rapid development of the industry, farming models are transitioning to intensive aquaculture [34]. However, frequent stressors such as high density and low dissolved oxygen under intensive culture conditions render *M. albus* susceptible to growth inhibition [35], oxidative stress, and immune suppression [36], which has become a critical bottleneck constraining the healthy development of the industry. Currently, research on the effects of berberine on enhancing economic returns through sustainable health management in an intensive *M. albus* culture remains unexplored. From the perspective of practical production applications, this study focuses on the effects of berberine on *M. albus* to investigate the putatively optimal dietary concentration range of berberine for promoting growth performance, antioxidant capacity, and hepatic metabolism in *M. albus* under intensive culture conditions. This research provides actionable insights for promoting healthy growth performance in intensively farmed *M. albus*, and promotes the industry’s upgrading toward green and efficient development.

## 2. Materials and Methods

All animal procedures detailed in this study were executed in strict conformity with protocols sanctioned by the Shanghai Academy of Agricultural Sciences Experimental Animal Ethics Committee (authorization code: SAASXM062438; sanction date: 7 August 2024).

### 2.1. Fish and Feeding Management

All *M. albus* used in this study were obtained from the Shanghai Academy of Agricultural Sciences and reared in plastic tanks (1.0 × 0.5 × 0.5 m). Six hundred domesticated healthy *M. albus* with uniform body sizes (Mean body weight: 25 ± 0.7 g; Initial biomass density: 2.5 kg/m^2^) were randomly selected from the stock population and allocated to four experimental groups: basal diet (43% crude protein, 7% crude fat; Hubei Zhaoliang Biotechnology Co., Ltd., Xiantao, China) supplemented with 0 mg/kg berberine (CON), 25 mg/kg berberine (BBR25), 50 mg/kg berberine (BBR50), and 100 mg/kg berberine (BBR100). The documented bitter taste of berberine hydrochloride, which reduces feed palatability and inhibits feed intake at elevated levels, caused 100 mg/kg berberine blunt snout bream (*Megalobrama amblycephala*) to spit out feed pellets due to bitterness [37]. *M. albus* is a strict carnivore with high sensitivity to feed freshness [34]; an upper limit of 100 mg/kg was imposed to avoid feed rejection. In crucian carp (*Carassius auratus gibelio*), 25 mg/kg represents the minimal threshold for detectable enzymatic inhibition [38], as sub-threshold doses elicit no significant effect. The 50 mg/kg level has been cross-species validated as the putatively optimal effective dose, significantly improving weight gain, feed intake, and hepatic health in blunt snout bream [37], and is widely recommended as the appropriate dietary inclusion level for fish. Collectively, the selected 0–25–50–100 mg/kg gradient encompasses a true zero control, a minimal threshold, a species-validated optimum, and a palatability-capped maximum, thereby enabling a robust effect of berberine in *M. albus*. Berberine chloride hydrate (purity ≥ 97%, CAS: 141433-60-5, Storage conditions: room temperature, dry, and sealed) was obtained from Shanghai Macklin Biochemical Technology Co., Ltd. (Shanghai, China), which was dissolved in water and thoroughly blended with the basal diet at graded concentrations (0, 25, 50, and 100 mg/kg), and the resulting experimental diets were fed to the fish maintained in the experimental tanks. Each treatment consisted of three replicates with 50 *M. albus* per tank, and the feeding trial lasted for 8 weeks. To maintain suitable water quality conditions, approximately two-thirds of the water volume was renewed daily (total ammonia nitrogen < 0.5 mg/L, dissolved oxygen ≥ 5.8 mg/L, pH 7.3 ± 0.2). The *M. albus* were fed once daily at 16:20 at a feeding rate of 2.5–3% of body weight.

### 2.2. Sample Collection and Analysis

Samples were collected on 27 September 2025. Upon completion of a 24 h deprivation of feed, five vigorous *M. albus* were arbitrarily chosen from every biological repetition (n = 3 tanks per treatment). These organisms were initially characterized as female individuals through gross anatomical inspection of surface features, with an age of eight months. The *M. albus* were anesthetized with MS-222 (Shanghai Experimental Reagent Co., Ltd., Shanghai, China). Under aseptic conditions, the samples were dissected and blood was collected via decapitation. The liver was excised using a sterilized scalpel. Liver samples from individual samples were stored in cryovials and snap-frozen in liquid nitrogen for subsequent liver enzyme activity and metabolomic analyses.

#### 2.2.1. Growth Indicators

We assessed growth indices according to these equations: weight gain rate (WGR, %) = 100 × (Wt − W0)/W0; survival rate (SR, %) = 100 × Nf/Ni; and feed conversion ratio (FCR) = feed intake (g)/body weight gain (g). The parameters indicate: W0 (g) as initial average body weight (IBW) per tank; Wt (g) as final average body weight (FBW) per tank; t (d) as the experimental duration; Ni as the starting fish number per tank; and Nf as the ending fish number per tank.

#### 2.2.2. Serum Biochemical Analysis

Whole blood was incubated overnight at 4 °C, followed by centrifugation at 3000 rpm for 10 min to separate serum for subsequent biochemical analyses. Spectrophotometric analyses at specific wavelengths served for multiple biochemical assessments: serum glucose via glucose oxidase–peroxidase (GOD-POD) chemistry (505 nm); hepatic phosphofructokinase (PFK) via coupled pyruvate kinase–lactate dehydrogenase (PK-LDH) monitoring NADH oxidation (340 nm); triglyceride (TG) via GPO-PAP (glycerol phosphate oxidase-peroxidase); total cholesterol (TC) via CHOD-PAP (cholesterol oxidase-peroxidase); high-density lipoprotein cholesterol (HDL-C) via phosphotungstic acid-magnesium (PTA-Mg) precipitation; and low-density lipoprotein cholesterol (LDL-C) via direct selective clearance. Additionally, phosphatase activities—alkaline phosphatase (AKP) and acid phosphatase (ACP)—were analyzed by the p-nitrophenyl phosphate (pNPP) method, while transaminase activities—alanine aminotransferase (ALT/GPT) and aspartate aminotransferase (AST/GOT)—were evaluated by the Reitman–Frankel method. These assays utilized commercially available kits (Nanjing Jiancheng Bioengineering Institute, Nanjing, China), and all procedures followed the manufacturer’s instructions precisely.

#### 2.2.3. Liver Enzyme Activity Analysis

Enzymatic activities were measured as follows: glutathione peroxidase (GSH-PX) via DTNB (5,5′-dithiobis-(2-nitrobenzoic acid) colorimetry, superoxide dismutase (SOD) via xanthine oxidase-WST-1 chemistry, catalase (CAT) via ammonium molybdate spectrophotometry, and lipid peroxidation (malondialdehyde, MDA) via thiobarbituric acid (TBA) reaction. Quantification relied on commercial assay kits (Nanjing Jiancheng Bioengineering Institute, Nanjing, China), with all procedures executed in strict adherence to the supplier’s manual.

#### 2.2.4. Untargeted Liquid Chromatography–Mass Spectrometry (LC-MS) Metabolomic Analysis

The extraction protocol comprised: (i) precisely weighing 30 mg of sample into 2 mL tubes with 200 µL ice-cold water and two stainless steel beads; (ii) dual homogenization cycles (55 Hz, 60 s each) using a high-throughput tissue grinder; (iii) adding 800 µL methanol–acetonitrile (1:1, *v*/*v*), sonicating for 30 min, and incubating at −20 °C for 45 min; (iv) centrifuging at 12,000 rpm and 4 °C for 20 min; (v) collecting 800 µL of supernatant for vacuum drying; (vi) reconstituting in 150 µL 50% methanol containing 5 ppm 2-chloro-L-phenylalanine (internal standard), vortexing for 30 s, and centrifuging at 12,000 rpm and 4 °C for 10 min; (vii) filtering through 0.22 µm membranes and centrifuging again at 12,000 rpm and 4 °C for 10 min; and (viii) transferring to autosampler vials. A quality control (QC) sample prepared by pooling 10–20 µL aliquots from each filtrate verified instrument stability.

Liquid Chromatography Conditions: An ACQUITY UPLC HSS T3 column (100 Å, 1.8 µm, 2.1 mm × 100 mm) served as the stationary phase at 40 °C, with the mobile phase delivered at 0.4 mL/min. Sample aliquots of 2 µL were injected via an autosampler held at 8 °C. The chromatographic runs utilized identical mobile phase compositions for both polarities—aqueous component containing 0.1% formic acid (eluent A) and acetonitrile component similarly acidified (eluent B). The stepwise elution program appears in Table S1.

Mass Spectrometry Conditions: High-resolution MS detection utilized an Orbitrap Exploris 120 configuration (version 4.7, Thermo Scientific, Waltham, MA, USA). Full-scan spectra were gathered in (DDA) format toggling between cationic and anionic modes. The (HESI) assembly maintained capillary thermal conditions of 320 °C and auxiliary heater settings of 300 °C, with nitrogen flows fixed at 40 arb (sheath) and 10 arb (auxiliary), and spray needle potentials established at 3.5 kV (positive mode) and −3.0 kV (negative mode). Full-scan mass spectra (MS1) were acquired at a resolution of 60,000 (at *m*/*z* 200) across the mass range of 70–1000 Da. The automatic gain control (AGC) target was set to standard levels, with a maximum ion accumulation time of 100 ms. For data-dependent acquisition, the four most abundant precursor ions were sequentially isolated for MS2 fragmentation employing higher-energy collisional dissociation (HCD) at 30% energy, 15,000 resolution, Standard AGC target, Auto Max IT, and a 4 s dynamic exclusion duration.

For non-targeted hepatic metabolomics, five individual liver samples per treatment were analyzed. All study samples and quality control (QC) samples underwent examination under the aforementioned chromatography–mass spectrometry settings. This study first derived an unbiased estimation of the total variance from the QC sample subset (Figure S1), followed by the evaluation of metabolite intensity consistency across injections using Pearson correlation coefficients (Figure S2). Raw data were Pareto-scaled and subsequently analyzed using univariate (volcano plot, *p* < 0.05 and |log_2_ fold change (FC)| > 1) (Figure S3) and multivariate statistical methods (variable importance in projection (VIP) > 1) to jointly screen for differential metabolites. To capture comprehensive metabolic signatures, we conducted screening in positive, negative, and dual-ion modes. The metabolic divergence between CON and BBR100 treatments was visualized and quantified via PCA and OPLS-DA. Those features exhibiting VIP > 1.0, fold change (FC) > 2 or <0.5, adjusted *p*-value (FDR) < 0.05 to control for false discoveries and statistical significance of *p* < 0.05 (two-tailed Student’s *t*-test) were flagged as differentially abundant metabolites and proposed as potential markers. Pearson correlation analysis was employed to evaluate covariation relationships among differential metabolites, with pairwise correlation coefficients (r) calculated for all differential metabolite abundances to construct a correlation matrix heatmap. We accessed the Kyoto Encyclopedia of Genes and Genomes (KEGG) resource for pathway analysis of differential metabolites covering both metabolic cascades and signal transduction processes. Mapping to KEGG (Release 115.0) pathways was performed via MetaboAnalyst 5.0. The 20 pathways with strongest enrichment significance (lowest *p*-values) were illustrated through bubble charts, and pathway-centric metabolic alterations were investigated utilizing Differential Abundance Score (DAS) diagrams. The DAS plots visualized differentially abundant metabolites within specific biochemical pathways to elucidate systematic metabolic shifts. These analyses were performed by Personalbio Technology Co., Ltd. (Shanghai, China).

### 2.3. Statistical Analysis

Statistical analyses were conducted using IBM SPSS Statistics (version 27.0; IBM Corp., Armonk, NY, USA), following initial data organization in Microsoft Excel (Microsoft Corp., Redmond, WA, USA). Levene’s test verified variance equivalency assumptions before parametric analyses. One-way analysis of variance (ANOVA) decomposed group-wise variability, while Tukey’s honestly significant difference (HSD) post hoc test traced significant pairwise separations. Values aggregated as mean ± standard deviation (SD), with statistical significance accepted at *p* < 0.05. For metabolomics and related omics data analysis, MetaboAnalyst 5.0 was utilized.

## 3. Results

### 3.1. Growth Performance

Enhanced growth performance manifested in the 25, 50, and 100 mg/kg berberine cohorts relative to untreated controls, encompassing elevated final weights, accelerated weight gain, and augmented survival rates (*p* < 0.05). Weight gain dynamics and survival percentages remained statistically equivalent between the 50 and 100 mg/kg concentration brackets (*p* > 0.05). Feed conversion ratios dropped precipitously across the berberine concentration gradient versus the CON group (*p* < 0.05), yet the 50 and 100 mg/kg levels yielded overlapping FCR distributions (*p* > 0.05) (Table 1).

### 3.2. Serum and Liver Biochemical Parameters

#### 3.2.1. Glucose Metabolism Parameters

**Figure 1:**
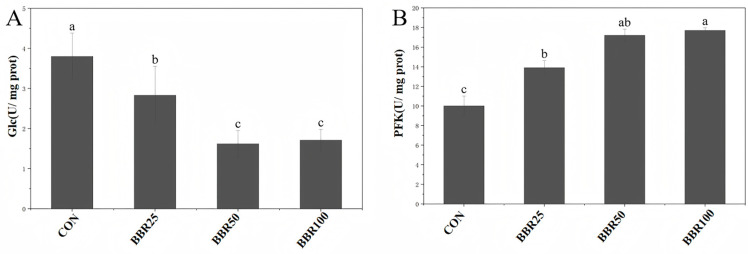
Glucose metabolism parameters contents in of *M. albus* among different concentrations of dietary BBR groups in different experimental groups. (**A**) Serum glucose (Glc). (**B**) Liver phosphofructokinase (PFK). The assignment of unique letters distinguishes significantly disparate group means (*p* < 0.05). CON, BBR 0 mg/kg group; BBR25: BBR 25 mg/kg group; BBR50: BBR 50 mg/kg group; BBR100: BBR 100 mg/kg group.

Serum glucose (Glc) levels declined significantly as berberine (BBR) concentration increased (*p* < 0.05), with the lowest values recorded in the BBR50 group. Glucose homeostasis remained statistically indistinguishable between the 50 and 100 mg/kg BBR concentration groups (*p* > 0.05). Phosphofructokinase (PFK) tracked positively with berberine concentration escalation (*p* < 0.05), plateauing at maximal activity in the 100 mg/kg treatment relative to control minima (Figure 1).

#### 3.2.2. Lipid Metabolism Parameters

**Figure 2:**
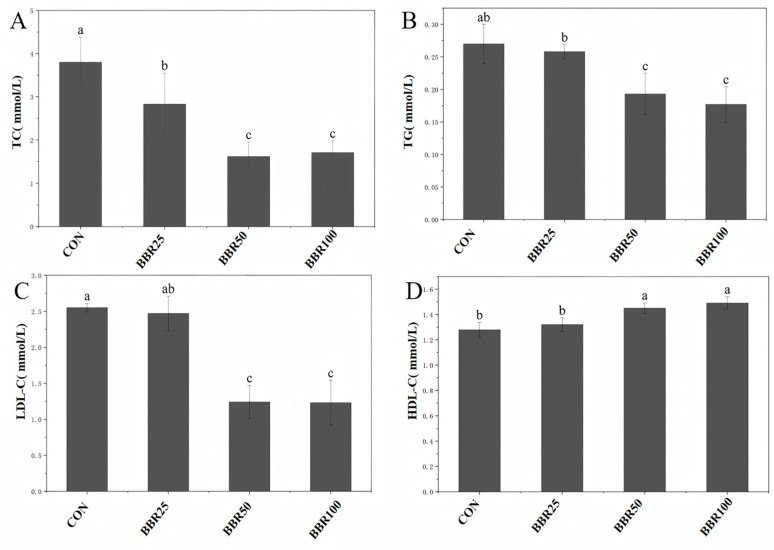
Lipid metabolism parameters contents of *M. albus* among different concentrations of dietary BBR groups in different experimental groups. (**A**) Total cholesterol (TC). (**B**) Triglyceride (TG). (**C**) Low-density lipoprotein cholesterol (LDL-C). (**D**) High-density lipoprotein cholesterol (HDL-C). Different letters indicate statistical significance (*p* < 0.05).

Berberine exerted multidirectional modulatory actions on the lipid metabolic profile of *M. albus* when compared with the CON group. While TC levels declined significantly under high BBR concentrations (*p* < 0.05), TG concentrations receded dose-proportionally (*p* < 0.05). LDL particles diminished at elevated BBR levels (*p* < 0.05), whereas HDL cholesterol traversed in the antagonistic direction (*p* < 0.05). No statistical divergence separated the BBR50 and BBR100 groups regarding lipid homeostasis (*p* > 0.05) (Figure 2).

#### 3.2.3. Serum Biomarkers of Liver Function

**Figure 3:**
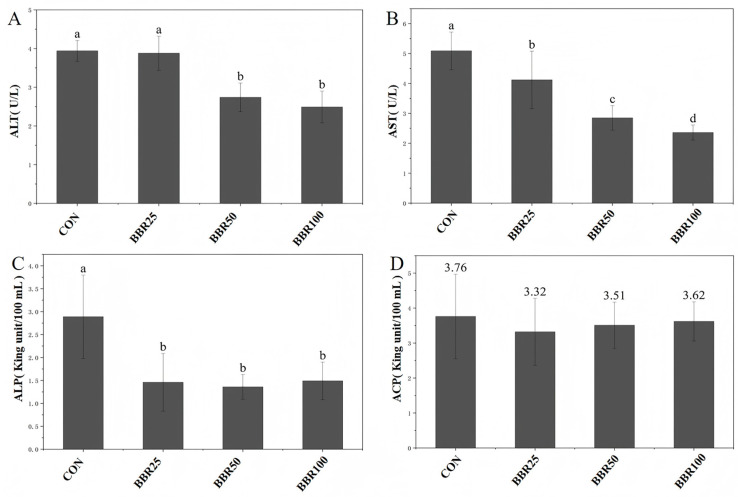
Serum biomarkers of liver function of *M. albus* among different concentrations of dietary BBR groups in different experimental groups. (**A**) Alanine aminotransferase (ALT). (**B**) Aspartate aminotransferase (AST). (**C**) Alkaline phosphatase (ALP). (**D**) Acid phosphatase (ACP). Superscript letter annotations signify probabilistic distinction between treatment means (*p* < 0.05).

As depicted in Figure 3, ALT levels showed a significant downward trend with escalating BBR concentrations (*p* < 0.05) and dose-escalation depressed AST activity most prominently (*p* < 0.05). ALP content dropped solely under high-BBR conditions (*p* < 0.05), in contrast to ACP parity across all arms (*p* > 0.05). Inter-group comparison revealed indistinguishable ALT and ALP values between the 50 and 100 mg/kg cohorts (*p* > 0.05).

#### 3.2.4. Liver Antioxidant Capacity Parameters

**Figure 4:**
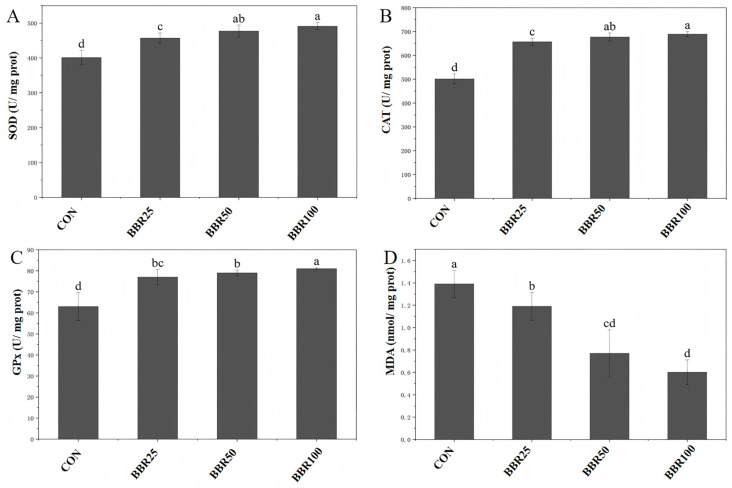
Liver antioxidant capacity parameters of *M. albus* among different concentrations of dietary BBR groups in different experimental groups. (**A**) Superoxide dismutase (SOD). (**B**) Catalase (CAT). (**C**) Glutathione peroxidase (GPx). (**D**) Malondialdehyde (MDA). Letter codes reflect distinct statistical groupings among treatment means (*p* < 0.05).

Results demonstrated that superoxide dismutase (SOD) activity increased significantly in a BBR concentration-dependent manner (*p* < 0.05). Catalase (CAT) activity was concomitantly upregulated (*p* < 0.05), and glutathione peroxidase (GPx) activity was also significantly enhanced (*p* < 0.05). MDA generation, the end-stage lipid peroxidation metabolite, diminished proportionally with incremental berberine supplementation (*p* < 0.05). The BBR100 cohort attained peak antioxidant enzyme capacity relative to other treatment groups (*p* < 0.05) (Figure 4).

### 3.3. Liver Metabolomics Analysis of *M. albus* Between the CON and BBR100 Groups

#### 3.3.1. PCA and OPLS-DA Assessment

**Figure 5:**
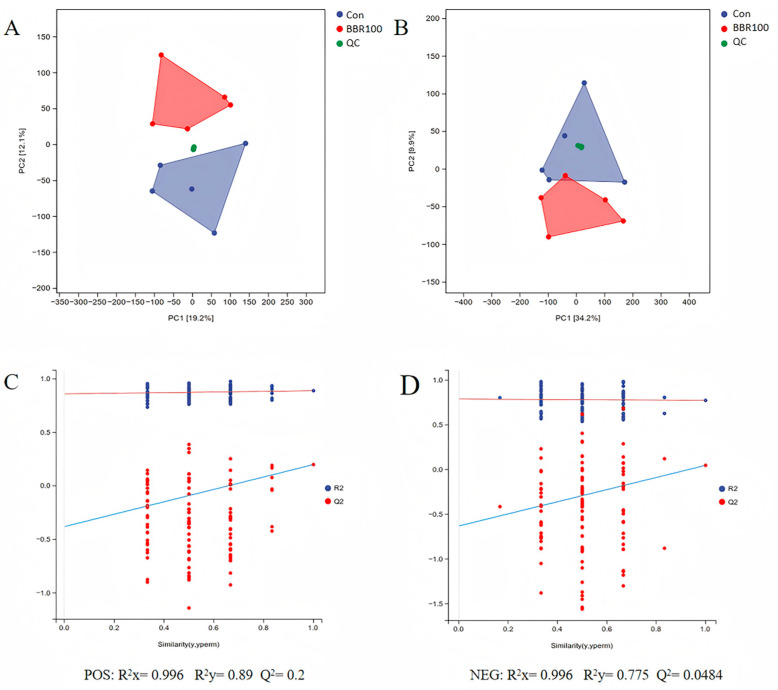
Pattern elucidation in liver metabolic profiling via multidimensional analytical modeling and cross-validation in *M. albus*. (**A**) Positive ion mode (ESI+) principal component analysis (PCA) score plots showing the distribution of control group (Con), berberine-treated group (BBR100), and quality control samples (QC). (**B**) Negative ion mode (ESI−) principal component analysis (PCA) score plots. Values in parentheses for PC1 and PC2 indicate the percentage of explained variance. (**C**) ESI+ mode OPLS-DA permutation test plots. (**D**) ESI− mode OPLS-DA permutation test plots, where blue and red dots represent R^2^ and Q^2^ values, respectively, and dashed lines indicate regression lines. Model parameters are annotated: R^2^X (explanatory power for X matrix), R^2^Y (explanatory power for Y matrix), and Q^2^ (predictive ability).

PCA revealed that, in the positive ion mode (ESI+), PC1 and PC2 explained 19.2% and 12.1% of the variance, respectively, with a cumulative contribution of 31.3% (Figure 5A). In the negative ion mode (ESI−), PC1 contributed 34.2% to the total variance, PC2 an additional 9.9%, summing to 44.1% cumulative coverage (Figure 5B). Under both ionization modes, the CON (blue) and BBR100 (red) cohorts traversed distinct regions of the multivariate space, with minimal overlap between clusters, whereas the quality control (QC) samples (green) were positioned intermediately between the two groups. OPLS-DA permutation testing indicated model parameters of R^2^X = 0.996, R^2^Y = 0.89, and Q^2^ = 0.2 for the ESI+ mode (Figure 5C), and R^2^X = 0.996, R^2^Y = 0.775, and Q^2^ = 0.0484 for the ESI− mode (Figure 5D). In both permutation tests, the Q^2^ intercept values were negative.

#### 3.3.2. Analysis of Differential Metabolites Based on OPLS-DA Models

**Figure 6:**
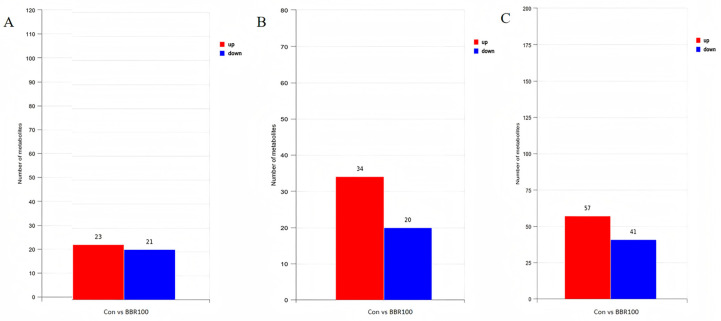
Statistical analysis of differential metabolites based on OPLS-DA models between the control group (CON) and berberine-treated group (BBR100) of *M. albus*. (**A**) Positive ion mode (ESI+). (**B**) Negative ion mode (ESI−). (**C**) Dual-mode merged differential metabolite counts. Chromatic coding distinguishes directional changes in metabolite abundance: warm hues designate elevated levels, and cool hues mark reduced expression.

OPLS-DA multivariate modeling demonstrated clear metabolic discrimination between experimental groups (Figure 6). Under positive ion mode (ESI+), 44 significantly altered metabolites (23 increased, 21 decreased) were identified (Figure 6A). Negative ion mode (ESI−) profiling revealed 54 differential features (34 upregulated, 20 downregulated) (Figure 6B). Subsequently, dual-polarity datasets were merged and duplicate annotations were removed, consolidating these findings into 98 distinct differential metabolites (57 upregulated, 41 downregulated) (Figure 6C).

#### 3.3.3. Integrated Metabolomic Analysis: Correlation, Pathway Enrichment, and Differential Abundance in *M. albus* Liver

**Figure 7:**
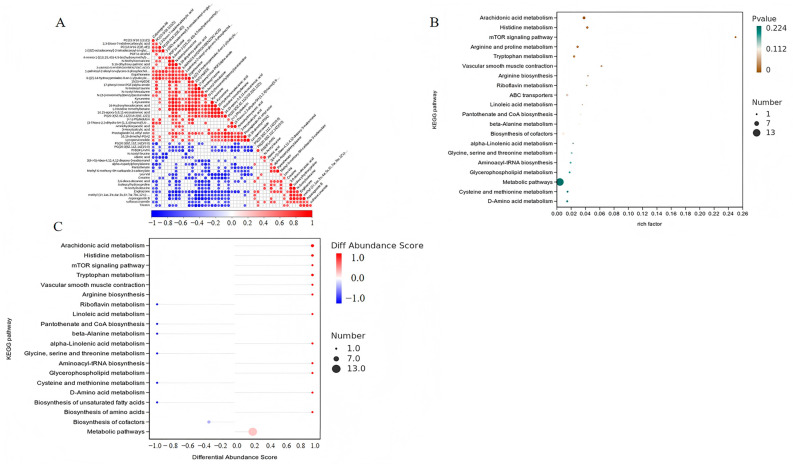
Correlation analysis of hepatic differential metabolites, KEGG pathway enrichment analysis, and differential abundance profiling of enrichment results between the control group (CON) and berberine-treated group (BBR100) of *M. albus*. (**A**) Correlation heatmap of differential metabolites displaying Pearson correlation coefficients (r) between metabolite abundances. Positive correlation coefficients (r > 0) map to warm chromatic tones, while inverse relationships (r < 0) correspond to cool hues; saturation levels track the absolute statistical magnitude. Metabolites are arranged via hierarchical clustering, with metabolite names displayed along the diagonal. (**B**) KEGG functional enrichment displayed via bubble plot geometry. Horizontal displacement (abscissa) scales with enrichment efficiency (rich ratio), vertical positioning (ordinate) catalogs pathway identifiers. Bubble area indicates the metabolite count per pathway; hue variations reflect enrichment (*p*-value). (**C**) Differential abundance analysis plot of enrichment results. The Y-axis shows metabolic pathway names, and the X-axis represents the differential abundance (DA) score. Intensified red coloration indicates pathway-wide expression trending toward upregulation, whereas intensified blue indicates expression trending toward downregulation.

To elucidate the underlying metabolic pathways associated with the observed metabolite alterations, pathway enrichment analysis employing the KEGG database was executed on differentially accumulated metabolites. Figure 7A presents the correlation patterns among these metabolites, revealing the intricate interconnections within the metabolic network that constitute the foundation for pathway-level interpretation. Figure 7B illustrates the enrichment significance of the identified pathways ranked by *p*-value, where bubble geometry encodes metabolite counts per pathway; color saturation indexes the corresponding enrichment significance. This visualization enables a comprehensive assessment of pathway-level perturbations, highlighting both the enrichment strength (rich factor) and the reliability of each pathway’s involvement.

As depicted in Figure 7B,C and detailed in Table S3, several metabolic pathways exhibited significant enrichment (*p* < 0.05). KEGG pathway enrichment analysis was ranked according to enrichment significance (*p*-value, ascending), with corresponding metabolites presented in Figure 7A and Table S3. Significantly upregulated pathways included arachidonic acid metabolism (metabolites: PC(15:0/18:1(11Z)), 14,15-epoxy-5,8,11-eicosatrienoic acid, and 5,6-DHET), histidine metabolism (ergothioneine and L-histidine trimethyl betaine), mTOR signaling pathway (L-arginine), arginine and proline metabolism (L-arginine), and tryptophan metabolism (L-kynurenine and xanthurenic acid) (*p* < 0.05) (Figure 7B,C).

## 4. Discussion

In our study, the improvement in growth performance, antioxidant capacity, and hepatic metabolism observed in the berberine-supplemented groups support its potential utility as a functional feed additive for *M. albus* under intensive culture conditions. Compared with the control group, BBR-treated groups exhibited significantly higher final weight, weight gain rate (WG), and survival rate (SR), consistent with recent findings in tilapia *(Oreochromis niloticus)* [32]. All BBR-treated groups demonstrated substantially lower FCR relative to controls, which directly confirmed the BBR enhancement of nutrient utilization and metabolic efficiency. Notably, there were no significant differences between BBR50 and BBR100 groups. In recent studies, blunt snout bream (*Megalobrama amblycephala*) [37] showed significant growth-promoting effects at a 50 mg/kg BBR feeding level. Juvenile black carp’s (*Mylopharyngodon piceus*) [39] growth performance parameters showed peak specifically at berberine concentrations of 98.26 and 196.21 mg/kg. In tilapia (*Oreochromis niloticus*) [40], a dietary introduction of 1 g/kg berberine elicited measurable enhancements in weight gain alongside improved FCR. In common carp (*Cyprinus carpio* L.) [41], the 200 mg/kg BBR inclusion level markedly enhanced feeding efficiency relative to other treatments. These studies indicate that the putatively optimal berberine concentrations for growth promotion vary considerably among different species, suggesting complex growth-promoting effects and the putatively optimal berberine inclusion level may be modulated by interspecific differences, developmental stages, rearing conditions, and experimental duration. Growth metrics remained statistically similar when comparing *M. albus* fed 50 mg/kg versus 100 mg/kg berberine, which means 50 mg/kg BBR may represent the putatively optimal physiological dose under the present experimental conditions, beyond which no additional growth benefits accrue.

To investigate the physiological mechanisms underlying the improved growth performance and feed utilization efficiency, subsequent analyses focused on serum biochemical parameters and liver function parameters. Graded BBR supplementation elicited concentration-dependent decrements in circulating glucose concentrations in *M. albus*, with nadirs apparent at 50 and 100 mg/kg dietary inclusion. This glucose-lowering trajectory coincided with elevated phosphofructokinase (PFK) activity in a concentration-linear fashion, tentatively suggesting that enhanced glycolytic throughput may represent a plausible, though not exclusive, contributing mechanism. Similarly, previous studies in aquatic organisms have demonstrated that BBR supplementation significantly lowered serum glucose while upregulating key glycolytic enzymes, such as phosphofructokinase (PFK), hexokinase (HK), and pyruvate kinase (PK) in crayfish (*Procambarus clarkii*) [42], largemouth bass (*Micropterus salmoides*) [43], and tilapia (*Oreochromis niloticus*) [32]. However, unchecked enhancement of glycolytic flux may carry a physiological cost. When glycolytic throughput exceeds the oxidative capacity of tissues, the terminal product pyruvate is diverted to lactate via anaerobic metabolism, resulting in lactate accumulation and localized tissue acidification [44]. Therefore, while the observed PFK activation in the present study suggests improved glucose clearance, it also raises the cautionary prospect that excessive anaerobic glycolysis could induce lactate accumulation and tissue acidosis, potentially impairing the long-term health of *M. albus* if the oxidative–glycolytic balance is not preserved. Furthermore, berberine (BBR) modulated the lipid metabolic profile of *M. albus*. While TC levels declined significantly under high BBR concentrations, TG showed a marked concentration-dependent reduction, accompanied by a significant decrease in LDL-C at higher doses and a reciprocal increase in HDL-C. Comparable findings have been reported in black sea bream (*Acanthopagrus schlegelii*) [45] and yellow catfish (*Pelteobagrus fulvidraco*) [46]. No significant differences in lipid parameters emerged between the BBR100 and BBR50 cohorts in the between-group analysis, suggesting metabolic benefits could saturate at moderate BBR supplementation levels (50 mg/kg), with no additional advantages conferred by doubling the dosage.

The decreased transaminase activities were suggestive of improved hepatic status in *M. albus*. Specifically, ALT levels exhibited a significant downward trend with escalating BBR concentrations, while AST demonstrated an even more pronounced dose-dependent decrease, whereas ALP content was significantly attenuated in the high-concentration BBR group, though ACP levels remained unresponsive to BBR supplementation. Similar observations have been reported in tilapia (*Oreochromis niloticus*) [26], common carp (*Cyprinus carpio* L.) [41], and channel catfish (*Ictalurus punctatus*) [47]. Comparable ALT and ALP values across BBR50 and BBR100 groups point toward a saturation threshold near 50 mg/kg, where liver-protective benefits cease to intensify despite increased dosing. Furthermore, a concentration-dependent enhancement of the antioxidant defense system was observed following BBR supplementation in *M. albus.* The enzymatic activities of SOD, CAT, and GPx showed marked elevation correlating positively with increasing BBR concentrations, with the BBR100 cohort displaying the maximal enzymatic activities among all experimental groups. Concomitantly, malondialdehyde (MDA) levels—an established biomarker of lipid peroxidation—showed a significant dose-dependent reduction, indicating attenuated oxidative damage. These results are similar to previous reports on crayfish (*Procambarus clarkii*) [42]. These findings suggest that BBR supplementation at 100 mg/kg optimizes the cellular redox balance in *M. albus* by bolstering the enzymatic scavenging capacity against reactive oxygen species (ROS). Collectively, although 50 mg/kg represents the “putatively optimal physiological dose” with the most favorable cost–benefit ratio, the 100 mg/kg supplementation level corresponded to the upper threshold of metabolic regulatory capacity within the current gradient. This higher dosage offered distinct investigative value regarding antioxidant defense and profound metabolic reprogramming, rendering it more suitable as the analytical target for non-targeted metabolomics to comprehensively elucidate the mechanistic underpinnings of berberine action. Therefore, the BBR100 group was chosen to perform non-targeted metabolomics.

Rigorous quality control (QC) procedures constitute the foundation of robust untargeted metabolomic investigations in *M. albus* hepatic tissue. Following the Metabolomics Standards Initiative (MSI) guidelines, an RSD threshold of <30% was established as the acceptable criterion for metabolomic data reliability [48]. In the present study, the dual-mode (ESI+ and ESI−) analyses achieved exceptional quality metrics with RSD values < 5% in both polarities (Table S2), well below the MSI benchmark. The QC total ion chromatogram (TIC) analysis confirmed reproducible and comprehensive metabolome coverage across both ionization modes (Figure S1), validating the robustness of our dataset for investigating berberine-induced metabolic reprogramming in *M. albus* liver.

The distinct chromatographic characteristics observed between ESI+ and ESI− modes reflect the complementary nature of dual-polarity metabolomics [49]. This orthogonal coverage is essential for comprehensive hepatic metabolic profiling, as ESI+ mode preferentially detects basic metabolites including phosphatidylcholines, amino acids, and nucleotides. Enhanced sensitivity toward acidic compounds—specifically bile acids, free fatty acids, and organic acids—is achievable through negative-polarity electrospray acquisition. This complementarity is critical for berberine research given its established effects on both lipid and bile acid metabolism [50].

Pearson correlation analysis of QC samples demonstrated near-perfect correlation coefficients (r = 1.00) among all QC pairs, indicating exceptional instrumental stability within analytical batches (Figure S2). Inter-group metabolic differences were assessed using multivariate methods comparing BBR100 against controls. Analytical reliability was confirmed through the tight clustering pattern observed for QC samples in PCA projections. According to metabolomic standards, an R^2^Y–Q^2^ difference exceeding 0.3 suggests potential model overfitting [51]; our ESI+ and ESI− models exhibited differences of 0.69 and 0.726, respectively, indicating limited predictive capacity for new samples despite adequate explanation of between-group variance. However, permutation testing validated model reliability with negative Q^2^ intercepts (blue Q^2^ lines below origin in Figure 5C,D), meeting the acceptance criterion [49]. The modest sample size (n = 5 per group) and inherent inter-individual variability in *M. albus* hepatic metabolism (evidenced by dispersion along PC2) likely contributed to the conservative Q^2^ values. Future investigations should incorporate expanded biological replication to achieve robust Q^2^ metrics.

Differential metabolite screening employed a stringent univariate–multivariate integrated strategy. We set the volcano plot discrimination thresholds at |log_2_FC| ≥ 1 and *p* < 0.05 to identify significantly altered candidates (Figure S3), ensuring statistical significance coupled with biological relevance [52]. Candidate features were retained in the OPLS-DA model only when VIP scores surpassed 1 and statistical testing yielded *p* < 0.05, thereby controlling false discoveries; VIP values quantify variable contribution to class discrimination, with values exceeding 1 indicating significant group separation [53]. Notably, merging dual-mode data yielded 98 differential metabolites—approximately twice the number identified in single-mode analyses. This expansion of detectable metabolites aligns with established metabolomic principles demonstrating that merged datasets significantly outnumber single-mode identifications [54,55,56], validating the necessity of dual-polarity approaches for comprehensive metabolic pathway elucidation.

The KEGG pathway enrichment analysis revealed significant enrichment of arachidonic acid (ARA) metabolism, histidine metabolism (ergothioneine synthesis), mTOR signaling, arginine/proline metabolism, and tryptophan metabolism in *M. albus* following berberine supplementation. Notably, ARA pathway derivatives including PC (15:0/18:1(11Z)), 14,15-epoxy-5,8,11-eicosatrienoic acid, and 5,6-DHET exhibited significant enrichment, implicating potential enhancement of eicosanoid biosynthesis and membrane phospholipid remodeling [57,58,59]. The accumulation of these metabolites could contribute to maintaining hepatocyte membrane integrity and fluidity, thereby facilitating efficient nutrient transport. Investigations into javeline goby (*Acanthogobius hasta)* nutrition revealed that hepatic intermediary metabolism responded markedly to graded arachidonic acid supplementation, with the inclusion level of 10.74 g ARA per kg total fatty acids eliciting peak specific growth performance [60]. In teleost fish, the dietary ARA dose determines the biological outcome: moderate eicosanoid levels may support immune homeostasis, whereas excess production triggers NF-κB-mediated hepatic inflammation, oxidative stress, and apoptosis [61]. The enrichment of histidine metabolism and arginine and proline metabolism with elevated ergothioneine is associated with protein synthesis [62,63,64]. Enhanced L-arginine concentration was concomitant with mTOR pathway activation, aligning with the conventional understanding of how specific amino acids facilitate protein synthesis enhancement in fish nutritional physiology [63], such as in abalone (*Haliotis discus hannai)* [65] and gibel carp (*Carassius auratus gibelio* CAS III strain) [66]. Furthermore, the accumulation of histidine metabolism with elevated ergothioneine and L-histidine trimethyl betaine is consistent with potent antioxidant activation, given that ergothioneine functions as a novel antioxidant synthesized via histidine trimethylation and cysteine conjugation in aquatic organisms [67]. In addition, the histidine–ergothioneine axis and tryptophan–kynurenine pathway may cooperatively influence oxidative stress and immune tolerance. Ergothioneine acts as a potent antioxidant with cytoprotective functions [67], and tryptophan metabolites via IDO/TDO enzymes regulate “immune tolerance mechanisms mediated by its metabolites” and counteract inflammatory responses [68]. These findings establish berberine not merely as a growth promoter, but as a metabolic stress resilience enhancer that reprograms hepatic lipid and amino acid metabolism toward a protective, anti-inflammatory phenotype. The saturating dose–response observed between 50 and 100 mg/kg suggests complex, species-specific pharmacokinetics, underscoring the necessity of precision dosing in aquaculture applications. Future investigations would elucidate the temporal dynamics of these metabolic shifts and validate the functional significance of key metabolites through targeted metabolite supplementation and pathway-specific inhibition studies. A limitation warrants acknowledgment. The 50 mg/kg putatively optimal dose requires empirical verification in other aquaculture species or different developmental stages of *M. albus*. Future investigations should be designed under a broader range of aquaculture conditions to determine the practical applicability and economic viability encompassing varying environmental stressors (temperature, hypoxia, stocking density) of berberine supplementation in intensive *M. albus* culture systems.

## 5. Conclusions

In conclusion, berberine supplementation was associated with alterations in lipid, amino acid, and signal transduction metabolite profiles, which are tentatively linked to membrane phospholipid remodeling and hepatocyte membrane integrity potentially favorable for nutrient transport and cellular homeostasis. Concurrently, these metabolic shifts implicate activated protein synthesis pathways, collectively suggesting a metabolic environment supportive of growth promotion. Simultaneously, it reinforced antioxidant defense and hepatoprotective effects in *M. albus*. Our findings establish 50 mg/kg dietary berberine as the putatively optimal physiological dosage for maximizing growth performance and feed utilization efficiency, whereas 100 mg/kg represented the upper threshold within the current gradient for triggering profound metabolic reprogramming and antioxidant defense without additional growth benefits. As a pilot study, several physiological and molecular parameters remain to be fully characterized. Future research should therefore evaluate the practical applicability and economic viability of berberine supplementation in intensive *M. albus* culture systems under diverse aquaculture conditions, particularly those involving key environmental stressors such as temperature, hypoxia, and stocking density. In addition, integrating metabolomics with transcriptomic and proteomic profiling will be essential to construct comprehensive regulatory networks linking berberine intake to hepatic lipid and amino acid metabolism.

## Figures and Tables

**Table 1:** Effects of different concentrations of dietary berberine on growth performance of *M. albus*.

Index	CON	BBR25	BBR50	BBR100
Initial weight (g)	24.66 ± 1.53	25.23 ± 0.70	24.90 ± 0.30	25.20 ± 0.55
Final weight (g)	34.83 ± 0.40 ^d^	38.6 ± 0.15 ^c^	46.36 ± 0.35 ^b^	49.03 ± 0.29 ^a^
WG (%)	136.4 ± 6.15 ^c^	152.3 ± 8.51 ^b^	184.0 ± 12.2 ^a^	196.7 ± 5.20 ^a^
FCR	5.512 ± 0.50 ^a^	4.071 ± 0.50 ^b^	2.84 ± 0.10 ^c^	2.59 ± 0.01 ^c^
SR (%)	82.00% ± 2.00 ^c^	86.00% ± 2.00 ^b^	92.00% ± 4.0 ^a^	92.00% ± 2.0 ^a^

Note: Data represent arithmetic means ± SD (standard deviation). Row-wise alphabetic notation denotes statistical disparity (*p* < 0.05). The dietary berberine supplementation levels were as follows: Control (CON), 0 mg/kg; BBR25, 25 mg/kg; BBR50, 50 mg/kg; and BBR100, 100 mg/kg. Abbreviations: weight gain rate (WG), feed conversion ratio (FCR), and survival rate (SR).

## Data Availability

We have provided the complete datasets in the published manuscript and the online Supplementary Materials.

## Supplementary Materials

The following supporting information can be downloaded at: https://www.mdpi.com/article/10.3390/life16050829/s1, Figure S1: Quality control (QC) total ion chromatogram (TIC) mass spectral peak comparison; Figure S2: Scatterplot matrix of metabolite intensity correlation among quality control (QC) samples; Figure S3: Univariate statistical analysis of differential metabolites in berberine-treated *M. albus* liver using volcano plots between of control group (CON) and berberine-treated group (BBR100); Table S1: Elution gradients for positive and negative ion modes; Table S2: Quality control sample total ion chromatogram intensity variation, positive and negative; Table S3: Quality control sample total ion chromatogram intensity variation in positive and negative.
