# Supplementary material for: Effects of Berberine on Growth Performance, Serum Biochemical Parameters, Hepatic Antioxidant Capacity and Metabolism in Monopterus albus
Source: Life (Basel). 2026 May 17;16(5):829. doi: 10.3390/life16050829 (PMC13208762; doi:10.3390/life16050829)
Supplement: Supplementary file 1 [file life-16-00829-s001.zip › life-4290264-supplementary.pdf]

Effects of Berberine on Growth Performance, Serum Biochemical Parameters, Hepatic Antioxidant Capacity and Metabolism in *Monopterus albus*

Xinran Tao [1,†], Weiwei Huang [2,†], Yifan Zhao [1], Muyan Li [1], Yuning Zhang [2], Hang Yang [2], Wenzong Zhou[2,*], and Mingyou Li [1,*]

[1] Key Laboratory of Exploration and Utilization of Aquatic Genetic Resources by the Ministry of Education, Shanghai Ocean University, Shanghai 201306, China; xxxholic98@163.com (X.T.); 17854232836@163.com (Y.Z.); limuyan0720@163.com (M.L.)
[2] Eco-Environmental Protection Research Institute, Ministry of Agriculture and Rural Affairs, Shanghai Academy of Agricultural Sciences, Shanghai 201403, China; hwwswx@163.com (W.H.); ynzhang@saas.sh.cn (Y.Z.); yhangqu2024@163.com (H.Y.); wwlv@saas.sh.cn (W.L.)
* Correspondence: zhouwz001@163.com (W.Z.); myli@shou.edu.cn (M.L.)
† These authors contributed equally to this work.

Supplements

Table S1. Elution gradients for positive and negative ion modes.

Time（min）	B%
0	5%
1	5%
4.7	95%
6	95%
6.1	5%
8.5	5%

Table S2. Quality control sample total ion chromatogram intensity variation in positive and negative. electrospray ionization modes.

Mode	TIC Intensity Range	Mean	SD	RSD	Evaluation
ESI+	$1.18\text{-}1.22\times10^{10}$	1.2025×10^{10}	0.017×10^{10}	1.4%	Excellent
ESI-	$2.80\text{-}3.01\times10^{10}$	2.9025×10^{10}	0.087×10^{10}	3.0%	Excellent

Table S3. KEGG pathway enrichment analysis of hepatic differential metabolites.

Pathway ID	Pathway	level	Trend	P value	Metabolite name
malb00590	Arachidonic acid metabolism	Lipid metabolism	up	0.002	PC (15:0/18:1(11Z), 14,15-epoxy-5,8,11-eicosatrienoic acid, 5,6-DHET
malb00340	Histidine metabolism	Amino acid metabolism	up	0.011	Ergothioneine, L-Histidine trimethylbetaine
malb04150	mTOR signaling pathway	Signal transduction	up	0.014	L-Arginine
malb00330	Arginine and proline metabolism	Amino acid metabolism	up	0.025	L-Arginine
malb00380	Tryptophan metabolism	Amino acid metabolism	up	0.034	L-Kynurenine, Xanthurenic acid
malb00564	Glycerophospholipid metabolism	Lipid metabolism	up	0.182	PC (15:0/18:1(11Z)
malb00591	Linoleic acid metabolism	Lipid metabolism	up	0. 183	PC (15:0/18:1(11Z)
malb01040	Biosynthesis of unsaturated fatty acids	Lipid metabolism	down	0.234	Docosahexaenoic acid
malb04270	Vascular smooth muscle contraction	Organismal Systems	up	0.056	14,15-epoxy-5,8,11-eicosatrienoic acid
malb00220	Arginine biosynthesis	Metabolism	up	0.082	L-Arginine
malb00740	Riboflavin metabolism	Metabolism	down	0.082	RIBOFLAVIN
malb02010	ABC transporters	Environmental Information Processing	up/down	0.085	L-Arginine, RIBOFLAVIN
malb00770	Pantothenate and CoA biosynthesis	Metabolism	down	0.102	Pantothenate
malb01240	Biosynthesis of cofactors	Metabolism	up/down	0.108	L-Kynurenine, RIBOFLAVIN, Pantothenate
malb00410	beta-Alanine metabolism	Metabolism	down	0.108	Pantothenate
malb00592	alpha-Linolenic acid metabolism	Metabolism	up	0.146	PC (15:0/18:1)
malb00260	Glycine, serine and threonine metabolism	Metabolism	down	0.158	Creatine
malb00970	Aminoacyl-tRNA biosynthesis	Genetic Information Processing	up	0.170	L-Arginine
malb00270	Cysteine and methionine metabolism	Metabolism	down	0.217	L-Methionine sulfoxide
malb00470	D-Amino acid metabolism	Metabolism	up	0.220	L-Arginine

Note: Pathway ID: Pathway identifier; Pathway: Pathway name; Trend: Number of up-/down-regulated metabolites enriched in the pathway.

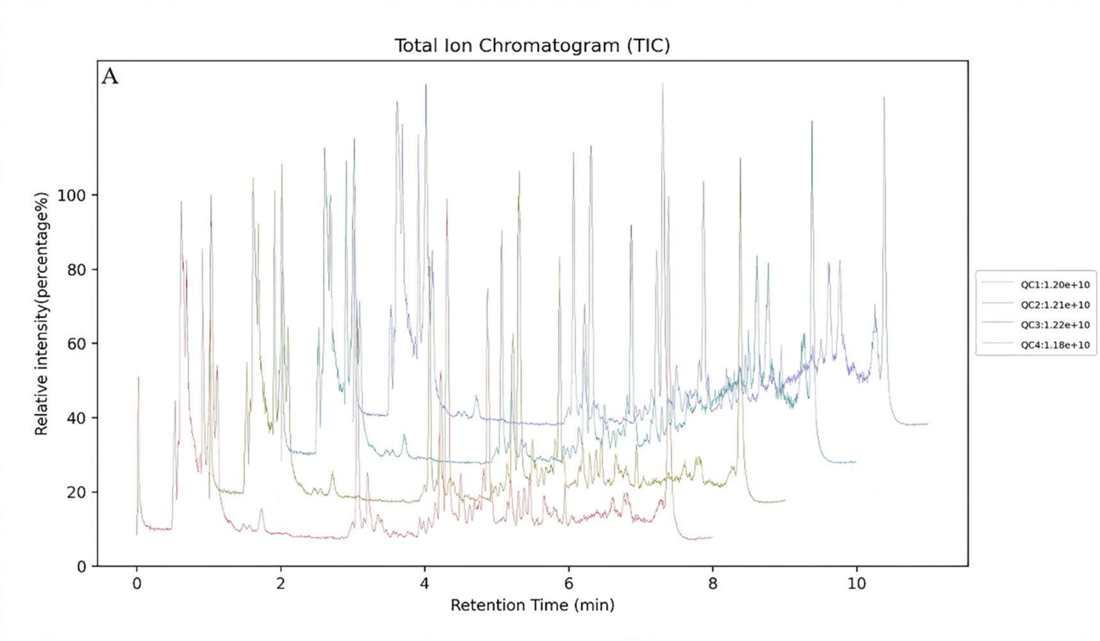

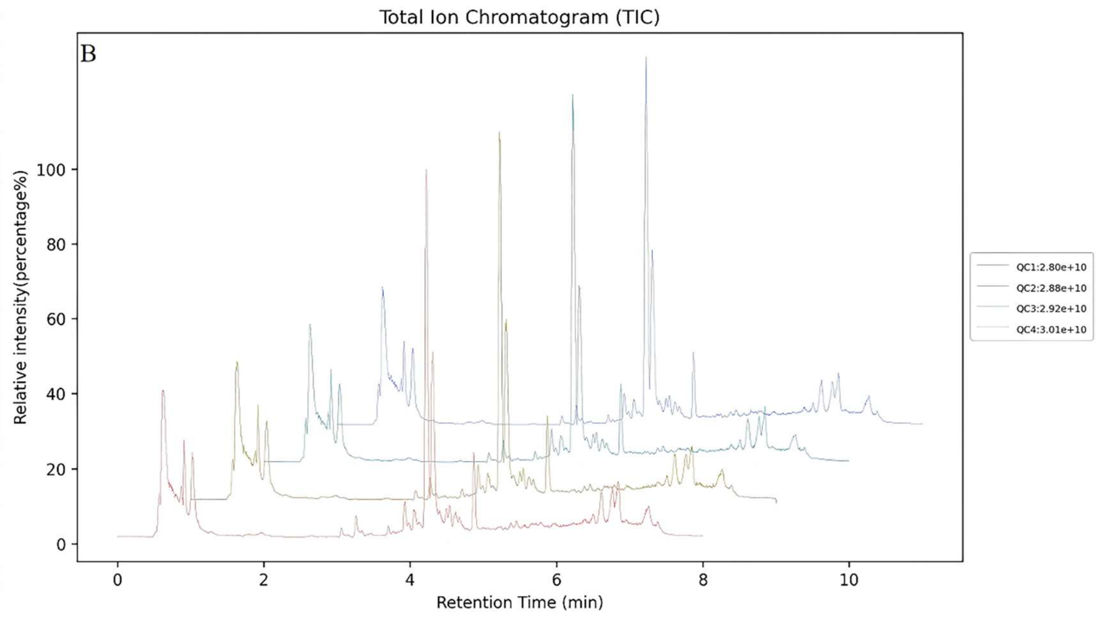

Figure S1. Quality Control (QC) Total Ion Chromatogram (TIC) Mass Spectral Peak Comparison. (**A**) Positive electrospray ionization (ESI+) mode. (**B**) negative electrospray ionization (ESI-) mode. The x-axis represents the retention time (min) of chromatographic peaks, and the y-axis represents the relative intensity (percentage of base peak). Total ion intensity values for each QC are labeled on the right side of the figure.

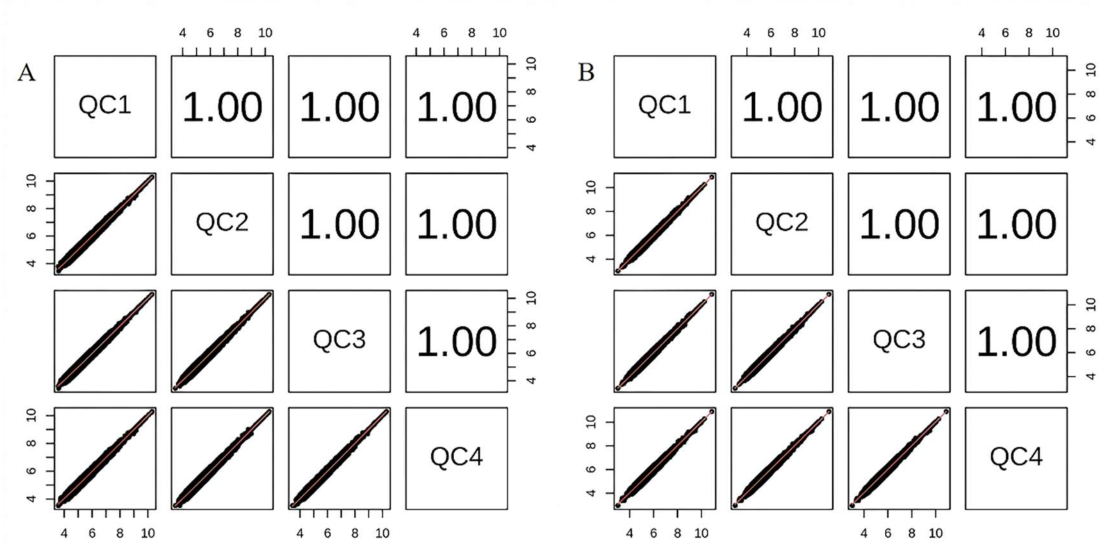

Figure S2. Scatterplot matrix of metabolite intensity correlation among quality control (QC) samples. (**A**) Positive electrospray ionization (ESI+) mode. (**B**) negative electrospray ionization (ESI-) mode. The diagonal panels display QC sample identifiers (QC1-QC4); lower triangular panels show pairwise scatterplots of log_2-transformed metabolite feature intensities; upper triangular panels display Pearson correlation coefficients (r).

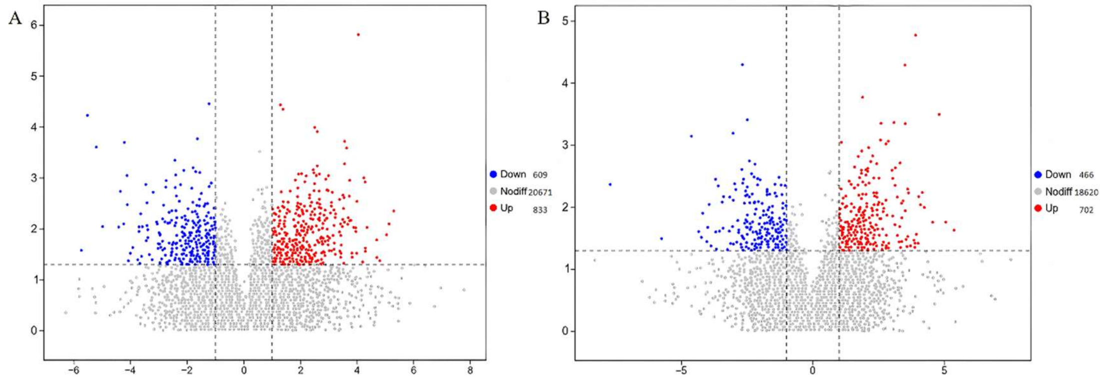

Figure S3. Univariate statistical analysis of differential metabolites in berberine-treated *M. albus* liver using volcano plots between of control group (Con) and berberine-treated group (BBR100). (**A**) Positive electrospray ionization (ESI+) mode. (**B**) negative electrospray ionization (ESI-) mode. Red points indicate upregulated metabolites, while blue points indicate downregulated metabolites.
